# Mental Illness Stigma and Associated Factors among Arabic-Speaking Religious and Community Leaders

**DOI:** 10.3390/ijerph18157991

**Published:** 2021-07-28

**Authors:** Klimentina Krstanoska-Blazeska, Russell Thomson, Shameran Slewa-Younan

**Affiliations:** 1 Mental Health, Translational Health Research Institute, School of Medicine, Western Sydney University, Sydney, NSW 2560, Australia; klimentinakrstanoskablazeska@gmail.com; 2 Centre for Research in Mathematics and Data Science, Western Sydney University, Sydney, NSW 2560, Australia; russell.thomson@westernsydney.edu.au; 3 Centre for Mental Health, Melbourne School of Population and Global Health, University of Melbourne, Melbourne, VIC 3053, Australia

**Keywords:** religious and community leaders, Arabic-speaking, refugees, stigma, mental illness

## Abstract

Evidence suggests that Arabic-speaking refugees in Australia seek help from informal sources, including religious and community leaders, when experiencing mental health issues. Despite their significant influence, there is scarce research exploring attitudes of Arabic-speaking leaders toward mental illness. The current exploratory study explored mental illness stigma and various factors among Arabic-speaking religious and community leaders. This study uses a subset of data from an evaluation trial of mental health literacy training for Arabic-speaking religious and community leaders. Our dataset contains the pre-intervention survey responses for 52 Arabic-speaking leaders (69.2% female; mean age = 47.1, SD = 15.3) on the ability to recognise a mental disorder, beliefs about causes for developing mental illness, and two stigma measures, personal stigma, and social distance. Being female was associated with a decrease in personal stigma. An increase in age was associated with an increase in personal stigma. Correct recognition of a mental disorder was associated with decreased personal stigma, and after adjusting for age and gender, significance was retained for the I-would-not-tell-anyone subscale. Endorsing the cause “being a person of weak character” was associated with an increase in personal stigma. There is an urgent need for future research to elucidate stigma to develop effective educational initiatives for stigma reduction among Arabic-speaking leaders.

## 1. Introduction

Arabic-speaking individuals represent a majority of the refugee population in Australia [1]. Indeed, Arabic was the top language spoken by humanitarian entrants who entered Australia between the years 2000 and 2014 with approximately 22% of humanitarian entrants speaking Arabic [2]. In the past five years, over half of the visas granted under Australia’s Refugee and Humanitarian Program were to individuals born in Iraq or Syria fleeing their country due to persecution, violence, and human rights violations [1]. Pre-migration experiences characterised by exposure to traumatic events and post-migration stressors place refugees at higher risk of developing mental disorders, particularly post-traumatic stress disorder (PTSD) and depression [3,4]. A systematic review of the literature on PTSD in Iraqi refugees resettled in Western countries revealed a prevalence rate ranging from 8% to 37.2% for PTSD and ranging from 28.3% to 75% for depression [5]. The elevated prevalence rate for developing mental disorders among refugees surpasses the general Australian population [6] and endures five years or longer after resettlement [7].

Despite elevated rates of mental illness and psychological distress, resettled refugees in Australia demonstrate low uptake of mental health services [8,9]. Instead, Arabic-speaking individuals and refugees demonstrate a preference for informal sources of help such as family, friends, and religious leaders [10,11,12]. Several factors are suggested to contribute to low levels of professional help-seeking. One key concept related to help-seeking behaviours and attitudes is mental health literacy (MHL) which is defined as: “knowledge and beliefs about mental disorders which aid their recognition, management or prevention” [13] (p. 182). Poor levels of MHL, particularly low levels of mental illness recognition and treatment knowledge consistent with Western biomedical models, have been reported in Arabic-speaking refugees resettled in Australia and their community leaders [9,14]. Notably, reduced levels of MHL have been associated with stigma [15]. Stigma may be conceptualised as an interaction of negative cognitions, emotional reactions, and behaviours [16]. Mental health-related stigma is especially prominent and problematic in Arabic-speaking communities [14,17].

Central to MHL are beliefs about the causes of mental illness. Although there is some evidence Arabic-speaking individuals endorse beliefs about mental illness consistent with biomedical models (e.g., chemical imbalance), supernatural and religious attributions of mental illness prevail in Arab society, even among medical students, paediatric hospital staff, and mental health professionals [18,19,20]. Stigmatising beliefs include mental illness originating from evil supernatural forces [21] or caused by a lack of faith and sin [14]. Stemming from the belief of the supernatural or higher-order origin of mental illness, seeking informal help from religious leaders or traditional healers is often the norm [14,22].

To understand the pervasiveness of stigmatising attitudes toward mental illness and help-seeking in Arab society, cultural influences of collectivism and the significance of the family unit must be considered. It has been posited that in collectivistic cultures, “non-normal” behaviour is more obvious and less tolerated within the community [23,24]. Members of the public tend to react with discriminatory behaviours towards people with mental illness (PWMI), such as a desire to distance oneself from those with mental illness to avoid public stigma [16]. High levels of reluctance to associate with PWMI across a range of social situations and relationships, including friends, teachers, neighbours, and family members have been well-documented in studies conducted with Arabic-speaking individuals [21,25,26,27].

The presence of mental illness in the family unit typically brings dishonour, damaging reputation and social standing [10,28]. In turn, family members are commonly discouraged from seeking professional help [14]. Mental illness is particularly stigmatising for women [29] and it is common for women to experience feelings of shame and embarrassment about seeking help outside the family [30]. However, it has been suggested Arabic-speaking females are more likely to seek counselling from professionals compared to males, consistent with findings from the general Australian community [31], in addition to religious assistance [14].

### 1.1. Arabic-Speaking Religious and Community Leaders

If help is sought for mental health problems, approaching religious and community leaders or traditional healers are often the first points of contact [14,32]. Religious leaders are swiftly available, willing to help individuals experiencing a crisis, and seeking their assistance is considered a less stigmatising and shameful action compared to seeking professional help [14]. Immigration and separation from traditional support may also prompt individuals who have migrated to new countries to seek greater affiliation with religious and community leaders [14,33].

General practitioners in the Australian Arab community reported leaders significantly influence whether an individual seeks professional mental health care such as from a psychiatrist or a general practitioner [14]. Despite their paramount influence, to our knowledge, only two studies exploring the attitudes of Arabic-speaking religious and/or community leaders toward mental illness. A Ph.D. dissertation comprising two published papers involving religious leaders in Sydney revealed a majority of leaders endorsed “drug/alcohol addiction” (93.5%) and psychosocial issues (91.2–93.5%) as important causes of mental illness [34]. However, religious causes were also rated as important (57.1–84.7%). Muslim leaders endorsed religious causes such as the “will of God” or “spiritual poverty” as more important causes for mental illness compared to Christian leaders [34]. One leader interviewed indicated a lack of confidence in the Western approach where the physical body is treated with medication often resulting in little improvement [14].

A recent study investigated Arabic-speaking Catholic male clerics’ attitudes regarding mental health in Lebanon [35]. A majority (84.9%) believed they would recognise a patient with mental illness. However, this was not objectively measured using a vignette methodology. Concerning causal beliefs regarding mental illness, “traumatic childhood” and “drug/alcohol addiction” were endorsed as most important and 70% agreed chemical imbalance is a causal factor. Despite a majority denying religious causes, 20–30% endorsed the notion that “spiritual poverty” and “demonic possession” are causes. Stigmatising attitudes endorsed by a majority of clerics equated a person with mental illness to a young child in terms of the control and discipline s/he requires, conveyed PWMI should have restrictions placed on their individual rights, and “are a burden on society”. Overall, the scarce research conducted to date seems to suggest Arabic-speaking leaders may hold stigmatising beliefs and attitudes, however, additional research is required. Moreover, an understanding of whether factors such as age and gender play a role in the stigmatising beliefs and attitudes toward mental illness held by Arabic-speaking leaders is important in developing targeted mental health promotion messages.

### 1.2. Current Study

The role of religious leaders in providing mental health care and facilitating help-seeking processes within immigrant communities is crucial [36,37,38]. To promote and facilitate the uptake of specialised mental health treatment for refugees, there is a critical need to explore stigma among Arabic-speaking leaders. If leaders have high levels of stigma themselves, this will negatively impact help-seeking and attitudes toward mental illness in the community. The aim of the current study is to explore relationships between measures of stigma and various factors in Arabic-speaking religious and community leaders. Drawing from the previous literature, it was hypothesised good MHL (i.e., correct problem recognition, knowledge of causes), will be associated with lower levels of stigma [32,39,40]. It was hypothesised being female and younger will be associated with lower levels of stigma [19,39,41].

## 2. Materials and Methods

### 2.1. Participants, Procedure, and Study Design

The present study is a subset of a dataset evaluating an MHL training course for Arabic-speaking religious and community leaders in South Western Sydney, Australia [42]. A total of 54 participants undertook a 6 h MHL training workshop (see Slewa-Younan and colleagues [42] for an overview of the intervention and power analysis). Of these individuals, 52 participants completed a self-report survey prior to and immediately following training but this study only utilised the pre-intervention survey responses. The training was advertised via religious centres and refugee service networks within South Western Sydney. Participants comprised volunteers who approached the workshop coordinator for enrolment. Individuals met eligibility criteria to participate if they were from an Arabic background, recognised themselves as religious or other community leaders, regularly communicated with Arabic-speaking refugee populations, and possessed a level of proficiency of the English language to ensure the understanding and the completion of the survey. The South Western Sydney Health Local District Research Ethics Committee (reference number 2019/ETH12040), jointly with Western Sydney University (H13411), provided approval of the research. Participants provided informed consent to participate in the study and for their data to be used in the research. Informed consent was obtained from participants when they attended the MHL training workshop and completed the pre-intervention survey. We would like to acknowledge that data from each participant in this study cannot be shared in order to comply with the protocols approved by the South Western Sydney Local Health District and Western Sydney University Human Research Ethics Committees.

### 2.2. Measures

The survey utilised in the current study is based on a survey developed by Jorm and colleagues [13] to assess MHL and has been adapted for refugee populations and utilised in a previous study by Slewa-Younan and colleagues [9]. Socio-demographic characteristics were also collected.

### 2.3. Recognition of PTSD as Dawood’s Main Problem

Previous studies have validated the use of a vignette for measuring MHL [43]. A culturally valid vignette portraying an Iraqi male refugee (“Dawood”) was used to assess participants’ ability to recognise Dawood’s mental health problem as PTSD. Dawood’s character met the 5th edition of the Diagnostic and Statistical Manual of Mental Disorders criteria for PTSD [44]. After participants read Dawood’s story, they were presented with an open-ended question: “What, if anything, do you think is wrong with Dawood?”. Open-ended responses correctly recognising Dawood’s character as having PTSD were coded as “Yes” if they mentioned any of the following wording: “PTSD”; “post-traumatic stress disorder”; “post-trauma/tic-stress/disorder”; and “PTS”. Responses that did not mention any of the aforementioned wording were coded as “No”.

### 2.4. Stigmatising Attitudes

Stigmatising attitudes toward mental illness were assessed via a personal stigma scale and social distance scale. The personal stigma scale assessed participants’ personal attitudes towards the character depicted in the vignette (“Dawood”) using statements adapted from an Australian National Survey [43,45]. Participants responded to seven statements assessing personal stigma using a 5-point Likert-style scale (1 = strongly disagree to 5 = strongly agree). The personal stigma scale was made up of three subscales: Dangerous/unpredictable, Weak-not-sick, and I-would-not-tell-anyone. The Dangerous/unpredictable subscale comprised items about Dawood’s dangerousness or unpredictability due to mental problems (e.g., “Dawood’s problem makes him unpredictable”). The Weak-not-sick subscale comprised items about the legitimacy and controllability of Dawood’s problems and the weakness of Dawood’s character (e.g., “Dawood’s problem is not a real medical illness”). The I-would-not-tell-anyone subscale comprised one item assessing if the participant would tell others if they had a problem like Dawood’s (“You would not tell anyone if you had a problem like Dawood’s”). The three subscales have been validated via structural equation modelling using data from a large Australian adult national survey [43] and utilised and validated in previous studies [46,47]. Higher scores for each subscale indicated higher levels of personal stigma.

The second measure of stigmatising attitudes toward mental illness comprised a Social Distance scale using statements developed by Link and colleagues [48]. The Social Distance scale assessed participants’ willingness to participate in various hypothetical relationships (e.g., neighbour, friend, colleague) with Dawood [48,49]. Participants were asked whether they would be happy: “to move next door to Dawood”; “to spend an evening socialising with Dawood”; etc. Participants responded to five statements assessing Social Distance using a 5-point Likert-style scale (1 = strongly disagree to 5 = strongly agree). A total Social Distance score was calculated by summing up the responses to each item. Higher scores indicated a greater desire for social distance.

### 2.5. Beliefs about Causes for Developing Mental Illness

Participants answered a question about possible causes of Dawood’s problem. Participants were asked: “How likely do you think each of the following is a factor in this sort of problem developing in anybody?”. Eleven causes were presented including “Punishment from God”, “Experiencing a traumatic event”, and “Being a person with a weak character”. Participants rated each item as “very likely”, “likely”, or “not likely”. For analysis purposes, responses for causes were recoded into 1 = “likely” (“likely” and “very likely” collapsed) and 0 = “not likely”.

### 2.6. Statistical Analyses

Statistical analyses were performed using the Statistical Package for Social Sciences (SPSS 26.0 for Mac, IBM Corp., Armonk, NY, USA). Firstly, a non-parametric Kendall’s Tau-b correlation was run to determine relationships between stigma scales. To test whether socio-demographic variables predicted stigma scale scores, standard multiple regression analyses with bootstrapping based on 1000 samples were performed with a stigma scale as the dependent variable and age and gender as independent variables. For the multiple linear regression models, percentage variance was presented based on R^2^. To measure the effect of stigma on the ability to correctly identify PTSD as Dawood’s main problem, four binary logistic regression analyses were undertaken with correct recognition of PTSD as the dependent variable and a stigma scale as the independent variable. Because age and/or gender predicted some stigma scales, logistic regression models were adjusted for age and gender thereafter.

To explore whether stigma affected the likelihood of endorsing a cause as “likely” for developing a problem like Dawood’s, a series of binary logistic regression analyses were performed. For each logistic regression analysis, a cause (e.g., “Punishment from God”) was entered as the dependent variable and a stigma scale (e.g., “Weak-not-sick”) was entered as the independent variable. Because age and/or gender predicted some stigma scales, logistic regression models were adjusted for age and gender thereafter. Logistic regression results were presented as odds ratios. A *p* < 0.05 was considered statistically significant.

## 3. Results

Socio-demographic characteristics of participants are presented in Table 1. A majority of the stigma scales were significantly correlated (Table 2). To reduce the risk of collinearity, separate regression analyses were conducted when exploring associations between each stigma scale and socio-demographic variables, correct recognition of PTSD, and endorsement of a cause. Means and standard deviations for stigma scale scores were: Weak-not-sick (M = 7.5; SD = 3.2); I-would-not-tell-anyone (M = 2.0; SD = 1.1); Dangerous/unpredictable (M = 6.2; SD = 2.4); Social Distance (M = 9.3; SD = 2.3).

### 3.1. Stigmatising Attitudes and Socio-Demographic Factors

Bootstrapping, a non-parametric resampling procedure, was used to test for statistical significance because the Kolmogorov–Smirnov test revealed the personal stigma subscales and Social Distance were not normally distributed (*p* < 0.05). For the regression analysis with Weak-not-sick as the dependent variable, gender (male = 1; female = 2) had a significant negative regression weight. Females were found to have lower Weak-not-sick scores compared to males, after controlling for age (Table 3). Age had a significant positive regression weight, indicating for a unit increase in age there was an increase in Weak-not-sick scores, after controlling for gender (Table 3). Age and gender explained 27.8% of the variation in the Weak-not-sick score. For the regression analysis with Social Distance as the dependent variable, bootstrapped coefficients revealed age had a significant positive regression weight, indicating for a unit increase in age there was an increase in Social Distance, after controlling for gender (*b* = 0.04, Bias = 0.00, Bootstrap SE = 0.02, 95% CI = 0.00, 0.08, *p* = 0.042). Age and gender explained 7.1% of the variation in the Social Distance score. Regression analyses with I-would-not-tell-anyone and Dangerous/unpredictable as dependent variables did not reveal significant differences. Age and gender explained 6.4% of the variation in the I-would-not-tell-anyone score. Age and gender explained 10.9% of the variation in the Dangerous/unpredictable score.

### 3.2. Stigmatising Attitudes and Recognition of PTSD as Dawood’s Main Problem

Fifty-one percent of participants correctly identified PTSD. Logistic regression models revealed significant results for personal stigma subscales, but not for Social Distance. For every single level increase in I-would-not-tell-anyone, the odds of correctly identifying PTSD decreased by a factor of 0.44 (95% CI = 0.23, 0.84, *p* = 0.013). For every single level increase in Weak-not-sick, the odds of correctly identifying PTSD decreased by a factor of 0.82 (95% CI = 0.68, 0.99, *p* = 0.039). For every single level increase in Dangerous/unpredictable, the odds of correctly identifying PTSD decreased by a factor of 0.77 (95% CI = 0.59, 0.99, *p* = 0.042). After adjusting for age and gender, significant results were retained for I-would-not-tell-anyone (Table 4).

### 3.3. Stigmatising Attitudes and Beliefs about Causes for Developing Mental Illness

The cause “being a person of weak character” was endorsed as “likely” by 43.1% of participants. Logistic regression analyses for the cause “being a person of weak character” and personal stigma subscales revealed significant results. For every single level increase in I-would-not-tell-anyone, the odds of rating “being a person of weak character” as a “likely” cause increased by a factor of 2.49 (95% CI = 1.26, 4.95, *p* = 0.009). Additionally, for every single level increase in Weak-not-sick, the odds of rating “being a person of weak character” as a “likely” cause increased by a factor of 1.45 (95% CI = 1.16, 1.80, *p* = 0.001). Finally, for every single level increase in Dangerous/unpredictable, the odds of rating “being a person of weak character” as a “likely” cause increased by a factor of 1.58 (95% CI = 1.17, 2.14, *p* = 0.003). After adjusting for age and gender, significant results were retained (Table 4).

## 4. Discussion

This preliminary study sought to explore mental illness stigma and associated factors among Arabic-speaking leaders. As expected, females were shown to have lower Weak-not-sick scores compared to males. An increase in age of one year was associated with an increase in Weak-not-sick and Social distance scores. In terms of problem recognition, a central component of good MHL, the odds of correctly identifying PTSD decreased for every single level increase in personal stigma. After adjusting for age and gender, the odds of correctly identifying PTSD decreased for every single level increase in the I-would-not-tell-anyone subscale only. Finally, the odds of selecting “being a person of weak character” as a cause increased for every single level increase in personal stigma.

Female leaders were predicted to have lower Weak-not-sick scores compared to male leaders. This gender difference in the Weak-not-sick subscale has been reported in the general Australian adult population [43]. Further, females compared to males, have been shown to have lower stigmatising attitudes in a Lebanese sample [39] and a Slovakian sample [50]. In contrast to the current finding, Arabic-speaking males have been shown to have more positive attitudes toward mental illness compared to females (see Zolezzi and colleagues [21] for a systematic review). Thus, our findings contribute to the existing mixed results among Arabic-speaking individuals which appear to be less conclusive than Australian-based research [41].

Weak-not-sick was the sole subscale revealing gender differences which may reflect the intersectionality of mental illness stigma and male gender roles in Arab society. The expression of negative emotional experiences may threaten a male’s capacity to perform their expected role as reliable and strong provider for the family unit [12]. Typically, disclosure of psychological problems is highly discouraged for males [12,51]. ‘Dawood’s’ symptoms appear to have affected his ability to provide for his family as he was portrayed as withdrawing from the lives of his children and questioning his life. In one Egyptian study, a female displaying symptoms of depression elicited more acceptance as a family member than a male [26]. Therefore, male gender roles and expectations in Arab culture may have influenced specific attitudes toward mental illness in the current study.

An increase in age was associated with increased Weak-not-sick scores and a higher desire for social distance, after controlling for gender. Older age has been associated with more stigmatising attitudes toward mental illness [50,52] and an increased desire for social distance [53]. Although preliminary, these findings are especially interesting as a previous study with male Arab Catholic clerics in Lebanon was unable to detect a significant relationship between age and attitudes toward PWMI [35]. A relationship between stigmatising beliefs about the causes of mental illness in Muslim clerics compared to Christian clerics has been demonstrated [34]. Although the current study did not explore the relationship between religion, country of origin, and stigma, the socio-demographic characteristics of the current study’s participants (e.g., majority of Muslim leaders) may have contributed to a difference in findings. This difference may also be related to our use of a vignette, allowing for a specific measure of personal stigma toward a character portrayed rather than general attitudes toward mental illness.

Correct recognition was associated with decreased levels of personal stigma. This is consistent with the finding that better attitudes toward mental illness was associated with more knowledge of mental illness in a Lebanese sample [39]. If a label of a specific disorder is placed, rather than placing a general label of “mental illness”, this may activate knowledge of specific treatments or steps to take to treat the illness and relate to beneficial attitudes [15]. To our knowledge, this is the first study to explore the relationship between the ability to recognise a specific mental disorder and stigma in Arabic-speaking individuals. Our preliminary findings indicate a necessity for future research to further investigate this complex relationship of stigma in Arabic-speaking individuals and across various mental disorders. Our finding also suggests initiatives aimed at reducing mental illness stigma should consider incorporating training to improve the ability to recognise specific mental disorders, as these concepts may be related.

Personal stigma was associated with a negative belief that being a weak person is a cause for developing a problem like Dawood’s. Similarly, the belief in a weak personality as a cause for various mental disorders was associated with higher scores on personal stigma in an Australian national survey [54]. Almost two-thirds of Iraqi individuals believed personal weakness is the cause of mental illness [55]. Endorsing weakness of character as a cause of mental illness is indicative of a dominant negative stereotype of mental illness and linked to low MHL [41,56]. This finding may be reflective of Arab cultural beliefs that link negative emotional expression with weakness, especially if such expression damages the family’s social standing and elicits public stigma [10,57].

Among refugee men, self-stigma for seeking help was a significant barrier to seeking help from not only formal sources but also from informal community sources [58]. If an individual chooses to first seek help from a leader, it is crucial leaders are aware of the vulnerabilities associated with seeking help [59]. The knowledge that such individuals are likely in the process of navigating their self-worth and identity in the context of experiencing a mental illness in a patriarchal society and strong adherence to masculine ideals should be imparted in any educational initiatives.

### 4.1. Limitations and Strengths

The current study’s limitations include a cross-sectional design which does not allow for causal explanations and the risk of social desirability as participants were leaders within the refugee community. Notably, the small sample size is a limitation in the current study, and future research with larger sample size is required to confirm the current findings. Participants were also individuals who actively sought MHL training. With regard to strengths, the use of a vignette allowed adaption to ensure a culturally sensitive measure to assess MHL. Research on MHL is primarily conducted using measures tapping into the Western biomedical conceptualisation of mental illness. Previous studies have resulted in conclusions of low MHL among culturally diverse populations including Arabic-speaking individuals despite lacking consideration of cultural perspectives on beliefs and treatment knowledge [12]. Evidently, the participants in our study reside in Western society. However, this does not entirely diminish the findings as contributing to our knowledge of MHL among culturally diverse populations.

### 4.2. Constraints on Generality

Arabic-speaking individuals are not a homogenous group and readers must keep the socio-demographic characteristics of the participants depicted in Table 1 in mind when generalising the findings. For example, our participants reside in Australia, a Western society, which may limit the generalisability of the current findings to Arabic-speaking leaders in Eastern societies. Further, refugee and humanitarian entrants in NSW have largely settled in the South Western Sydney local health district meaning the leaders in the current study may have higher exposure to refugees compared to leaders in other local health districts in NSW [60].

The results of the current study likely depend on the materials used. Our measure of MHL and stigmatising attitudes was limited to one vignette depicting a married male who is a father experiencing a specific mental disorder, PTSD. Readers should be cautious about generalising the findings as stigmatising attitudes and mental health literacy may vary depending on the character depicted in the vignette. For example, gender has been reported to moderate mental illness stigma [61]. Future research should utilise vignettes that depict individuals of various socio-demographic characteristics and types of mental illness. The findings of such studies will further inform specific target areas for reducing stigma through educational initiatives for Arabic-speaking leaders.

### 4.3. Clinical Implications

The mental health beliefs of the leaders in our study may represent a challenge to treatment adherence. Psychologists are encouraged to adopt a multilevel approach and collaborate with the client’s family, religious, and community leaders. Psychologists should be willing to reach out to leaders within their client’s community to develop insight into and a shared understanding of their client’s worldview. Psychologists should be aware of the limitations of Western-based models underpinning cognitive-behavioural interventions when applied to Arabic-speaking individuals [62]. Such Western-based models may be different from the conceptualisations of mental illness Arabic-speaking refugees and their leaders possess. Challenging cognitive distortions and faulty beliefs posited to maintain symptoms of mental illness are core tools used in cognitive-behavioural therapy [63]. However, if clients are exposed to mental health beliefs from their leaders such as an experience of depression represents a test in life, an opportunity to cleanse and become more religious, as noted by a leader in Youssef and Deane’s [14] study, challenging this thinking is likely to mismatch the client’s worldview and they may feel misunderstood. Rather than challenging such beliefs, psychologists should be sensitive to these issues and deliver interventions that work alongside how Arabic-speaking refugee communities view and respond to mental illness, encompassing their cultural and religious beliefs.

## 5. Conclusions

Our preliminary findings lay the groundwork for future research by exploring factors associated with stigma among Arabic-speaking religious and community leaders. Leaders have immense potential to influence help-seeking and foster helpful attitudes toward mental illness. Our findings indicate an urgent need for future larger-scale research to further elucidate stigma, thereby, refining stigma reduction initiatives among Arabic-speaking leaders.

## Figures and Tables

**Table 1:** Socio-demographic Characteristics.

Characteristics	Pre-Training (*n* = 52) *	
	*N*	Valid *%*
Gender		
Male	16	30.8
Female	36	69.2
Age (M, SD)	47.1 (15.3)	
Country of Origin (top 3)		
Iraq	18	33.3
Australia	13	24.1
Lebanon	8	14.8
Organisations represented by participants		
Churches/Mosques	17	32.7
Non-government organisation	21	40.4
Government organisation	14	26.9
Language spoken at home (top 3)		
Arabic	39	72.2
English	7	13
Assyrian	2	3.7
Marital Status		
Never married	7	13.7
Married	35	68.6
Fiancée/partner	2	3.9
Divorced	6	11.8
Widowed	1	2
Education		
High school	3	5.6
Certificate	5	9.3
Diploma	5	9.3
Bachelor	31	57.4
Masters	6	11.1
For those born overseas		
Years in Australia (M, SD)	17.6 (10.9)	
Arrival status in Australia		
Refugee	7	19.4
Migrant	29	80.6
Religion		
Muslim	34	65.4
Christian	17	32.7

* Due to missing data may not add to 52.

**Table 2:** Kendall’s Tau-b Correlations of Personal Stigma Subscales and Social Distance Scale.

Variable	I-Would-Not-Tell-Anyone	Weak-Not-Sick	Dangerous/Unpredictable
I-would-not-tell-anyone			
Weak-not-sick	0.43 ***		
Dangerous/unpredictable	0.50 ***	0.34 ***	
Social distance	0.18	0.20	0.33 **

*** *p* < 0.001; ** *p* < 0.01 (2-tailed).

**Table 3:** Bootstrap coefficients for multiple linear regression model of socio-demographic predictors of Weak-not-sick subscale, with 95% percentile confidence intervals. Confidence intervals and standard errors are based on 1000 bootstrap samples.

Model	*B*	Bias	SE	*p*	95% CI (Lower, Upper)
Constant	7.27	−0.07	2.25	0.003	2.97, 12.00
Gender	−2.06	−0.01	0.91	0.036 *	−3.86, −0.08
Age	0.08	0.00	0.03	0.011 *	0.03, 0.13

*Note. n* = 50 due to missing data; *B =* Bootstrap regression coefficient; Bias = Bootstrap bias estimate; SE = Bootstrap standard error; CI (lower, upper) = confidence interval (lower limit, upper limit). * *p* Value is significant.

**Table 4:** Odds ratios of eight logistic regression analyses of predictors of correct recognition of PTSD and endorsement of a cause as “likely”, adjusted for age and gender.

Variables	Problem Recognition (Dependent Variable)	Cause (Dependent Variable)
Personal stigma subscales and Social distance scale (independent variables)	Correct recognition of PTSD (95% CI lower, upper)	“being a person of weak character” (95% CI lower, upper)
I-would-not-tell-anyone subscale Weak-not-sick subscale Dangerous/unpredictable subscale Social Distance scale	0.48 * (0.25, 0.94) 0.88 (0.71, 1.10) 0.83 (0.63, 1.10) 1.00 (0.77, 1.29)	2.52 * (1.17, 5.44) 1.32 * (1.03, 1.68) 1.46 * (1.06, 2.02) 0.99 (0.76, 1.30)

*Note.* Only causes that yielded statistically significant results are presented; Regressions presented are adjusted for age and gender; CI lower, upper = confidence interval lower limit, upper limit for odds ratio. * *p* < 0.05.

## Data Availability

We would like to acknowledge that data from each participant in this study cannot be shared in order to comply with the protocols approved by the South Western Sydney Local Health District and Western Sydney University Human Research Ethics Committees.
